# Impact of Attendance at Flipped Classroom Sessions on Renal Pathophysiology Scores

**DOI:** 10.7759/cureus.108754

**Published:** 2026-05-12

**Authors:** Danilyn M Angeles, Lawrence K Loo

**Affiliations:** 1 Basic Sciences, Loma Linda University School of Medicine, Loma Linda, USA; 2 Internal Medicine, Loma Linda University School of Medicine, Loma Linda, USA

**Keywords:** active learning strategies, flipped classroom method, preclinical medical students, quality improvement study, renal pathophysiology

## Abstract

Introduction: Various teaching strategies emphasizing active learning have been used to enhance student engagement, but the impact of these strategies on student performance is not well-documented. This quality improvement project assessed whether non-mandatory, in-person flipped classroom sessions on renal physiology influenced renal pathophysiology exam scores during the renal-urinary block and explored students' attitudes toward these sessions. Additionally, we aimed to explore students' attitudes toward attending these non-mandatory flipped sessions.

Methods and results: The study involved 180 sophomore medical students, 73 of whom voluntarily attended flipped sessions, while 107 did not. Our primary finding was that students who participated in flipped sessions had significantly higher end-of-block exam scores, with a dose-response relationship observed among those attending all sessions. Fewer students failed the block, and more achieved high-pass scores among participants who attended all flipped sessions. Secondary findings were that students who attended the flipped classroom sessions also performed better on both lower-order and higher-order cognitive multiple-choice questions. Student feedback on flipped sessions was mixed. Some thought that they were great for learning and wanted more, while others cited concerns about the number, scheduling, and preparation time required.

Conclusion: While the data suggest an association with the flipped sessions and improved end-of-block performance, the lack of randomization leaves room for confounding factors. Higher scores could reflect unmeasured student characteristics, such as prior knowledge, academic background, time management skills, motivation, and a preference for active learning. To clarify the relationship between active learning strategies, classroom attendance, and performance, future studies should employ prospective randomized designs.

## Introduction

Renal physiology is inherently challenging and complex. Surveys of internal medicine residents and medical students consistently report that nephrology is one of the most difficult physiology courses during their preclinical years [1-3]. The term “nephrophobia” has been coined to describe the aversion trainees feel toward renal physiology [4], which may contribute to empty classrooms, lack of student engagement, and superficial learning. This aversion likely stems not only from the difficulty of the subject but also from challenges faced by both students and instructors in applying and integrating basic science principles, particularly physiology, into clinical contexts.

To enhance student interest in nephrology, new methods emphasizing active learning have been utilized. These methods include web-based modules [5], simulation [6], use of models or drawings [7-9], digital chalk-talk videos [3], class discussion of clinical cases [10], and board games [11]. While these methods have shown promise in improving student attitudes toward nephrology, their impact on learning outcomes, such as knowledge retention and transfer, remains largely unmeasured, and the approaches themselves demonstrate considerable methodological diversity and statistical heterogeneity. While student attitudes toward nephrology seemed to have improved due to the use of these newer teaching strategies, Steinert et al. noted that the subjective response of students ("I like it") does not consistently correlate with objective measures of learning [12]. While assessing student reactions to teaching methodologies is important, it is crucial to ascertain whether learning has occurred, how it has influenced behavior, and ultimately, how these behavioral changes contribute to the system, for example, enhancing patient care [13].

We conducted this quality improvement project to assess whether attendance and participation in non-mandatory flipped classroom sessions focused on renal physiology impacted renal pathophysiology exam scores. The flipped classroom method is an established active learning strategy known to enhance student learning and performance among health professionals [13-15]. This method entails students dedicating time outside of class to prepare using pre-recorded videos, handouts, and assigned readings. During in-person class time, students engage in question-and-answer sessions, small group discussions, problem-solving, and critical thinking exercises [14]. Given the importance of understanding normal kidney function as a foundation for studying kidney dysfunction, we sought to evaluate whether attending non-mandatory in-person sessions in renal physiology, delivered using a flipped classroom format, could enhance performance on renal pathophysiology.

## Materials and methods

This quality improvement project occurred in 2022 at a large medical school (Loma Linda University School of Medicine, Loma Linda) in the western United States. The study qualified for exempt status by the university’s Institutional Review Board. Two years before the project was conducted, the medical school underwent a major revision in its four-year curriculum, transitioning from a discipline-based to an organ-based curriculum. In this new curriculum, faculty members were encouraged to utilize active learning methods, such as team-based learning and a flipped classroom.

The renal urinary block is a 3 ½ week course taught in the second year of the new curriculum. Table 1 shows the distribution of lecture content during the block, divided by disciplines. Each discipline has its own set of lecturers, so the instructors for renal physiology are different from those for renal pathophysiology. Topics specific to renal physiology are listed in Table 2. Five topics were delivered in a flipped classroom format, while four were delivered through traditional in-person lectures. Students were encouraged, but not required, to attend the flipped classroom sessions.

**Table 1:** Renal-urinary block lecture content

	Lecture Hours
Renal Anatomy (Embryo, Histology)	4
Renal Physiology	9
Renal Pathophysiology	23
Renal Pathology	6
Renal Pharmacology	4
Others (microbiology, lifestyle medicine)	2

**Table 2:** Renal physiology topics

Traditional Lecture Format	Flipped Classroom Format
Renal circulation	Glomerular filtration
Extracellular fluid volume regulation	Solute and water transport along the nephron
Extracellular fluid osmoregulation	Mechanisms of Urinary Concentration and Dilution
Kidney metabolism	Potassium homeostasis
	Renal regulation of acid-base balance

For the flipped classroom sessions, students were provided a pre-recorded video of the topic at least 48 hours before the session, as well as handouts and slides. Students were asked to preview the material before class. During the in-person session, the same instructor (DA) utilized 10-12 audience response questions using PollEverywhereä to assess the student’s understanding of the material, explain difficult concepts and clarify misconceptions of important physiological mechanisms. Most questions required critical thinking and analysis, and integrated material from previous organ blocks. Students were given the time and the option to discuss the question with a classmate before they turned in a response. For the traditional lecture format, the same instructor (DA) delivered the lecture in-person using traditional PowerPoint slides over a 50-minute period. The traditional lecture was recorded and provided to the students by the end of the day. Physiology content was delivered during the first three weeks of the course: the majority during the first week (seven hours) and the rest during weeks 2 and 3. Pathophysiology content was delivered by a different group of physician instructors a few days after the relevant physiology material. Formative assessments of student learning were performed weekly, and a final summative examination made up of multiple-choice questions was given at the end of the block. The final multiple-choice examination consisted of 96 multiple choice questions (MCQ), 39 of which were renal pathophysiology questions. Each MCQ was crafted by the lecturer who conducted the session, ensuring alignment with the session's learning objectives. The instructors who wrote the final exam questions for physiology are different from those who wrote the final exam questions for pathophysiology. None of the pathophysiology instructors attended the flipped classroom sessions and the session was not recorded. An examination review committee that consists of content experts and exam creation experts reviewed and approved all questions used for summative assessments.

Statistical analysis

Data analysis was conducted using IBM SPSS Statistics for Windows, Version 29 (Released 2022; IBM Corp., Armonk, New York, United States) with the level of significance set at α = 0.05. Continuous data were summarized using means with standard deviations and analyzed with an independent t-test and analysis of variance (ANOVA) when the data are normally distributed. Multiple comparisons were further adjusted with Bonferroni tests to adjust for inflated type 1 error. Median with minimum and maximum values is used to summarize the continuous variables and further analyzed using Mann-Whitney and Kruskal-Wallis tests for non-parametric data. Categorical data were reported using frequency and percentages and analyzed with chi-square or Fisher's exact test.

## Results

Demographics

There were 180 sophomore medical students enrolled in the renal-urinary block. A total of 73 (40%) students voluntarily attended the flipped classroom sessions, and 107 (60%) students did not. There were no significant differences in age, ethnicity, gender, and MCAT scores between those who attended and those who did not attend the flipped sessions (Table 3).

**Table 3:** Demographics of students who attended versus did not attend flipped sessions All values represent number and percentage except where indicated with median (minimum – maximum) and mean + SD. Test used chi-square except where indicated with *Mann-Whitney test or #Independent samples t-test. SD = standard deviation. df: degrees of freedom.

	No (N=107)	Yes (N=73)	P-value	Test Statistic	df	Effect Size
Age (Median (Min–Max))	25 (20–38)	25 (22–34)	0.209	U = 3719	—	r = 0.09*
Ethnicity			0.138	χ² = 6.97	4	V = 0.20
Asian/Pacific Islander	42 (65%)	23 (35%)				
Black Non-Hispanic	11 (52%)	10 (48%)				
Hispanic	20 (77%)	6 (23%)				
White Non-Hispanic	25 (50%)	25 (50%)				
Multiple Ethnicities	9 (50%)	9 (50%)				
Gender			0.442	χ² = 0.59	1	V = 0.06
Male	48 (56%)	37 (44%)				
Female	59 (625)	36 (38%)				
MCAT (Mean ± SD)	510 ± 5.6	511.4 ± 6.2	0.103	t = -1.63	178	d = 0.25^#^

Final pathophysiology exam scores in relation to attendance at flipped classroom sessions

The final MCQ exam has 39 pathophysiology questions. The average score for the pathophysiology MCQs is 71.4 ± 10.6%. We found that students who attended the flipped classroom sessions had significantly higher average scores (73.9% ± 9.02) than those who did not attend (69.8% ± 11.2) (Table 4). The average pathophysiology score of those who attended all of the flipped classroom sessions was significantly higher than that of those who did not attend any sessions (76.9% ± 8.0 vs. 69.8% ± 11.4, P = 0.033, effect size 0.39) (Table 5). As expected, the students who voluntarily attended the flipped sessions had significantly higher final exam scores compared to those who did not attend, since pathophysiology exam questions comprised 40% of the final exam.

**Table 4:** Pathophysiology exam scores of students who attended versus did not attend flipped sessions *Mann-Whitney Test, +Cohen’s d

	Attended Flipped Sessions (N=73)	Did Not Attend Flipped Sessions (N=107)	*P-value	^+^Effect Size
Pathophysiology exam score (%), mean ± SD	73.9 ± 9.02	69.8 ± 11.2	0.010	0.39

**Table 5:** Number of flipped sessions attended in relation to pathophysiology exam scores *indicates significance at an alpha of < 0.05; significant difference observed between groups "0" and "5" using the Kruskal-Wallis test

Number of Flipped Sessions Attended	Pathophysiology Score
0 (N = 107)	69.8 ± 11.4
1 (N=15)	68.9 ± 7.7
2 (N=6)	76.9 ± 10.6
3 (N=16)	73.1 ± 7.9
4 (N=11)	73.4 ± 10.3
5 (N=25)	76.9 ± 8.0*

Scores on higher-order cognitive skills MCQs compared to scores on lower-order cognitive skills MCQs in relation to attendance at flipped classroom sessions

Since all our MCQs and learning objectives are aligned with Bloom’s taxonomy, we examined the relationship between students’ scores on higher-order cognitive MCQs and the number of flipped sessions attended. Our focus was on promoting higher-order cognitive skills, such as analysis, application, and synthesis, over lower-order cognitive skills like recall and comprehension [15,16]. These higher-order skills are crucial for passing state and national certifying exams and for the practical application of medicine. We found that students who voluntarily attended the flipped sessions had significantly higher scores in both lower-order and higher-order MCQs (Table 6). We also found that those who voluntarily attended all five flipped sessions had significantly higher lower-order and higher-order scores than those who did not attend any of the flipped sessions (Table 7).

**Table 6:** Attendance to flipped session and average scores on higher-order versus lower-order multiple choice questions (MCQs) Lower-order MCQs = 52, Higher-Order MCQs = 41; *Mann-Whitney Test; + Rank biserial correlation: small effect: ⏐r⏐≈ 0.1; medium effect: ⏐r⏐≈ 0.3; medium effect: ⏐r⏐≈ 0.5

	Attended Flipped Sessions		P-value*	⏐r⏐^+^
	Yes (N=73)	No (N=107)		
Lower-order MCQ scores (%)	79.6 ± 8.6	74.9 ± 10.8	0.004	0.3
Higher-order MCQ scores (%)	77.7 ± 10.6	73.8 ± 12.3	0.041	0.2

**Table 7:** Number of flipped sessions attended and scores on higher-order versus lower-order multiple choice questions (MCQs) *indicates significance at an alpha of < 0.05; significant difference observed between groups "0" and "5" using the Kruskal-Wallis test

Number of Flipped Sessions Attended	Scores on “Higher Order” Cognitive Skills MCQs	Scores on “Lower Order” Cognitive Skills MCQs
0 (N = 107)	73.8 ± 12.2	74.9 ± 10.8
1 (N=15)	70.1 ± 13.1	75.4 ± 9.1
2 (N =6)	79.3 ± 7.36	76.9 ± 8.1
3 (N=16)	78.3 ± 8.2	78.8 ± 8.9
4 (N=11)	76.3 ± 8.6	79.7 ± 9.3
5 (N=25)	82.3 ± 9.6*	83.2 ± 7.7*

Distribution of students to the criterion-based grading scale and attendance to the flipped classroom sessions

Although the multiple-choice examination contributed only 70% to the final grade, while the other 30% was based on team-based learning scores, quizzes, and patient inquiry assignments, we examined how attendance at flipped classroom sessions altered the number of students in each grade category, based on the MCQ exam alone. The grade categories are: Not Pass (< 69.6%), Marginal Pass (69.6 - 70%), Pass (70.1 -80.0%) and High Pass (> 80%). As shown in Table 8, attendance at flipped classroom sessions was associated with a lower percentage of students in the “Not Pass” category and a higher percentage of students in the “High Pass” grade category.

**Table 8:** Final multiple choice question grade scale comparing flipped classroom attendance (0 versus 5 sessions)

Grade Scale	0 Sessions (n=107)	5 Sessions (n=25)	P-value	Fischer’s Exact Test statistic	df	Effect Size (Cramer’s V)
			0.016	6.97	3	0.28
Not Pass (< 69.6%)	31 (29.0%)	3 (12.0%)				
Marginal Pass (69.6 - 70%)	4 (3.7%)	0 (0.0%)				
Pass (70.1 – 80.0%)	36 (33.6%)	5 (20.0%)				
High Pass (> 80.0%)	35 (33.6%)	17 (68.0%)				

Student feedback on flipped classroom sessions

Student feedback on the flipped classroom format was mixed, with many highlighting the challenge of preparing for multiple flipped sessions within a single week. To address this, students suggested either spreading the sessions more evenly across the schedule or reducing their total number to make them more manageable. Conversely, some students expressed a desire for additional flipped sessions, reflecting varying preferences within the group.

## Discussion

Our data showed that students who voluntarily attended flipped classroom sessions in renal physiology had significantly higher renal pathophysiology exam scores than medical students enrolled in the renal urinary block (Tables 4, 5). Notably, the average score of students who did not attend the flipped sessions was 69.8%, which would be a failing grade if the final grade were based solely on renal pathophysiology. Because the MCAT scores were not significantly different in those who attended versus those who did not attend the flipped sessions (Table 3), we do not believe the self-selected students who attended the optional flipped classrooms were “smarter” (based on standardized testing) than those who chose not to attend. However, MCAT scores are not fully predictive of future performance in medical school. Students who voluntarily attended non-mandatory sessions might differ from those who did not. There may be differences in motivation, time management skills, a preference for active engagement, or level of commitment and effort to academic success, all of which could affect exam scores [17]. Likewise, students who did not attend non-required in-person sessions may have different levels of self-efficacy and the ability to self-regulate compared to those who attended the mandatory sessions [18]. 

Because the instructors who wrote the exam questions for physiology were different from those who wrote the exam questions for pathophysiology, we do not believe that attendance at flipped sessions “gave the exam away” or compromised the integrity of the exam. Our data suggest that additional exposure to the material, through self-study before the sessions and engaging with practice questions alongside peers and faculty, enhanced students' understanding of renal pathophysiology concepts, improved knowledge retention, and fostered critical thinking, ultimately leading to measurable improvements in their final grades [18]. Because pathophysiology exam questions comprised most of the questions in the final exam, attending the flipped classroom sessions decreased the students’ chance of failing the course and increased the students’ chance of receiving “High Pass” scores (Table 8). Using the Kirkpatrick model for educational outcomes, we believe we have demonstrated not only improved retention of actual learning (level 2) and a change in student learning behaviors (level 3) [13].

We also observed a dose-response effect, where students who attended more flipped sessions tended to have higher scores than those who attended fewer sessions. More importantly, those who attended all five sessions had significantly higher final block exam scores and final pathophysiology scores and demonstrated a better understanding of both lower-order and higher order thinking, compared to those who did not attend any flipped sessions (Tables 5, 7). To our knowledge, this dose-response relationship between classroom attendance at flipped sessions and improved exam performance has not been previously described and further supports the concept that increased exposure to the material using an active learning approach may contribute to higher scores [19].

The flipped classroom represents an "active learning" approach, embodying a broader shift in medical education away from traditional lecturing ("sage on the stage") towards a more collaborative and interactive model ("guide on the side”) [20]. This shift aligns with Malcolm Knowles' principles of andragogy (teaching adults) as opposed to pedagogy (teaching children) [21]. In the flipped classroom method, students first study the material independently and then engage in quizzes or other retrieval practice activities, a technique known to enhance long-term retention compared to simple restudying [22]. However, the efficacy of active learning methods, such as the flipped classroom, relies heavily on student preparation before the session and presence in the classroom, where they actively participate in critical thinking, clinical problem-solving, collaborative learning, and receive feedback [23] while dialoguing with peers and the instructor.

Nevertheless, attendance in non-mandatory in-person sessions has been declining in medical schools. In our study, only 40% of the class attended the flipped sessions, a trend similar to findings by Gardner et al., where nearly half of the students (43%) never attended non-mandatory lectures during the second year of medical school [24]. Kursweil et al. reported even lower attendance, with only 5% participation in non-mandatory neuroscience and behavior block sessions [25]. Various factors contributed to this decline, including the availability of recorded lectures, access to supplemental resources, perceived lecture quality, and scheduling flexibility issues [24]. Moreover, a recent study indicated that 63% of students begin preparing for Step 1 during their sophomore year, further reducing the time available for attendance to non-mandatory sessions [26]. These considerations are vital in designing a curriculum that provides students both the knowledge and experience with proven learning strategies that foster self-directed and lifelong learning [27].

We gained valuable insights from students' end-of-block comments about the flipped sessions. Students' responses to the optional flipped classroom are varied, with some expressing a desire for more of these sessions, while many others voiced concerns about the extra time and effort required for preparation. The latter illustrates the caution about using learner reaction as the sole measure of the effectiveness of an educational intervention [12]. These reactions echo findings from prior research, which consistently indicate that many students opt against evidence-based, effective learning strategies due to what's termed the "misinterpreted-effort hypothesis" [28]. This hypothesis suggests that students perceive the additional effort associated with proven learning methods (like the flipped classroom) as indicative of poor learning [29]. In other words, there's a belief that if learning isn't easy or requires minimal effort, it must not be an intelligent or effective study strategy. Unfortunately, utilizing study strategies that require the lowest mental effort (e.g., rereading or underlining) can negatively impact learning, since such strategies are often ineffective [30]. Nevertheless, student feedback prompted us to adjust the scheduling of flipped sessions to allow students more time to prepare for these sessions.

Because students were not randomly assigned to flipped or traditional sessions, the study is subject to residual confounding and selection bias. Since students self-selected to attend the optional flipped classroom sessions, they may have possessed characteristics or factors, such as interest in the subject, self-regulation, motivation, or time management skills that influenced both their decision to attend and their academic performance. Nevertheless, the integrity of the exam was maintained, as the physiology questions were developed by one instructor, while pathophysiology questions were written by a separate group of instructors. A prospective randomized controlled trial examining the effect of the flipped classroom method on renal pathophysiology scores is required to definitively answer this question.

## Conclusions

Despite these limitations, our study provided valuable data and insights that informed our decisions on balancing the number and type of required versus voluntary sessions and optimizing session schedules to enhance student learning and support effective lifelong learning strategies. Although this was not a randomized study, our quality improvement project found that attending in-person classes using the flipped classroom method is associated with improved student exam performance. Future prospective randomized studies are needed to firmly establish the causality of these findings.

## References

[1] Prasad C, Sanger S, Chanchlani R, Kirpalani A, Noone D (2022). Engaging medical students and residents in nephrology education: an updated scoping review. J Nephrol.

[2] Jhaveri KD, Sparks MA, Shah HH (2013). Why not nephrology? A survey of US internal medicine subspecialty fellows. Am J Kidney Dis.

[3] Roberts JK, Sparks MA, Lehrich RW (2016). Medical student attitudes toward kidney physiology and nephrology: a qualitative study. Ren Fail.

[4] Williams SE (2018). Nephrophobia: teaching renal medicine to the undergraduate student. J Nephrol.

[5] Lawless ME, M.S. M.S., Henriksen J, Raab S, Dintzis S, Rendi MH (2014). Modular education systems improves pathology education in renal and genitourinary systems. Lab Investigation.

[6] Tena-Santana G, Guiterrez-Morales I, Navvaro-Gilabert A (2014). A simulated practical scenario to teach students the pathophysiological manifestations of a renal insufficiency. Acta Physiol.

[7] Giffen ZC, Carvalho H (2015). Development of a manipulative for nephron physiology education. Adv Physiol Educ.

[8] Bhaskar A, Oommen V (2018). A simple model for demonstrating the factors affecting glomerular filtration rate. Adv Physiol Educ.

[9] Robinson PG, Newman D, Reitz CL, Vaynberg LZ, Bahga DK, Levitt MH (2018). A large drawing of a nephron for teaching medical students renal physiology, histology, and pharmacology. Adv Physiol Educ.

[10] Dietz JR, Stevenson FT (2011). Active learning in a large medical classroom setting for teaching renal physiology. Adv Physiol Educ.

[11] Zakaria F, Wan Zukiman WZHH, Shah A (2020). “U-R-INE’ town board game: learning AKI the fun way!!. Kid Internal Rep.

[12] Steinert Y, Mann K, Centeno A, Dolmans D, Spencer J, Gelula M, Prideaux D (2006). A systematic review of faculty development initiatives designed to improve teaching effectiveness in medical education: BEME Guide No. 8. Med Teach.

[13] Kirkpatrick DL, Kirkpatrick JD (2006). Evaluating Training Programs: The Four Levels. https://books.google.co.in/books?hl=en&lr=&id=BJ4QCmvP5rcC&oi=fnd&pg=PR5&dq=Evaluating+training+programs:+the+four+levels&ots=Mp_6b8xZ3Y&sig=WkgiRmhEge8O3IdWBwoRqIy2FmM&redir_esc=y#v=onepage&q=Evaluating%20training%20programs%3A%20the%20four%20levels&f=false.

[14] Persky AM, McLaughlin JE (2017). The flipped classroom - from theory to practice in health professional education. Am J Pharm Educ.

[15] Anderson LW, Krathwohl DR (2025). A Taxonomy for Learning, Teaching, and Assessing: A Revision of Bloom’s Taxonomy of Educational Objectives. https://books.google.com/books/about/A_Taxonomy_for_Learning_Teaching_and_Ass.html?id=EMQlAQAAIAAJ.

[16] Billings MS, Hussie K, Kulesher A (2016). Constructing Written Test Questions for the Basic and Clinical Sciences. https://www.nbme.org/educators/item-writing-guide.

[17] Richardson M, Abraham C, Bond R (2012). Psychological correlates of university students' academic performance: a systematic review and meta-analysis. Psychol Bull.

[18] Kauffman CA, Derazin M, Asmar A, Kibble JD (2018). Relationship between classroom attendance and examination performance in a second-year medical pathophysiology class. Adv Physiol Educ.

[19] Guyatt GH, Oxman AD, Kunz R, Vist GE, Falck-Ytter Y, Schünemann HJ (2008). What is "quality of evidence" and why is it important to clinicians?. BMJ.

[20] King A (1988). From sage on the stage to guide on the side. College Teaching.

[21] Knowles MS, Holten III EF, Swanson RA, Robinson PA (2020). The Adult Learner: The Definitive Classic in Adult Education and Human Resource Development. 6th ed.

[22] Carpenter SK, Pan SC, Butler AC (2022). The science of effective learning with spacing and retrieval practice. Nat Rev Psychol.

[23] Lu C, Xu J, Cao Y (2023). Examining the effects of student-centered flipped classroom in physiology education. BMC Med Educ.

[24] Gardner G, Feldman M, Santen SA, Mui P, Biskobing D (2022). Determinants and outcomes of in-person lecture attendance in medical school. Med Sci Educ.

[25] Kurzweil D, Swanberg M, Meyer E (2022). A pragmatic approach to flipping the classroom for 170 medical students. Int J Design Learning.

[26] Burk-Rafel J, Santen SA, Purkiss J (2017). Study behaviors and USMLE step 1 performance: implications of a student self-directed parallel curriculum. Acad Med.

[27] Mylopoulos M, Brydges R, Woods NN, Manzone J, Schwartz DL (2016). Preparation for future learning: a missing competency in health professions education?. Med Educ.

[28] Macaluso JA, Beuford RR, Fraundorf SH (2022). Familiar strategies feel fluent: the role of study strategy familiarity in the misinterpreted-effort model of self-regulated learning. J Intell.

[29] Kirk-Johnson A, Galla BM, Fraundorf SH (2019). Perceiving effort as poor learning: The misinterpreted-effort hypothesis of how experienced effort and perceived learning relate to study strategy choice. Cogn Psychol.

[30] Dunlosky J, Rawson KA, Marsh EJ, Nathan MJ, Willingham DT (2013). Improving students' learning with effective learning techniques: promising directions from cognitive and educational psychology. Psychol Sci Public Interest.

